# In Vitro Influence of Mycophenolic Acid on Selected Parameters of Stimulated Peripheral Canine Lymphocytes

**DOI:** 10.1371/journal.pone.0154429

**Published:** 2016-05-03

**Authors:** Maciej Guzera, Lidia Szulc-Dąbrowska, Anna Cywińska, Joy Archer, Anna Winnicka

**Affiliations:** 1 Department of Pathology and Veterinary Diagnostics, Faculty of Veterinary Medicine, Warsaw University of Life Sciences, Warsaw, Poland; 2 Department of Veterinary Medicine, University of Cambridge, Cambridge, United Kingdom; 3 Department of Preclinical Sciences, Faculty of Veterinary Medicine, Warsaw University of Life Sciences, Warsaw, Poland; Institut National de la Santé et de la Recherche Médicale (INSERM), FRANCE

## Abstract

Mycophenolic acid (MPA) is an active metabolite of mycophenolate mofetil, a new immunosuppressive drug effective in the treatment of canine autoimmune diseases. The impact of MPA on immunity is ambiguous and its influence on the canine immune system is unknown. The aim of the study was to determine markers of changes in stimulated peripheral canine lymphocytes after treatment with MPA *in vitro*. Twenty nine healthy dogs were studied. Phenotypic and functional analysis of lymphocytes was performed on peripheral blood mononuclear cells cultured with mitogens and different MPA concentrations– 1 μM (10^−3^ mol/m^3^), 10 μM or 100 μM. Apoptotic cells were detected by Annexin V and 7-aminoactinomycin D (7-AAD). The expression of antigens (CD3, CD4, CD8, CD21, CD25, forkhead box P3 [FoxP3] and proliferating cell nuclear antigen [PCNA]) was assessed with monoclonal antibodies. The proliferation indices were analyzed in carboxyfluorescein diacetate succinimidyl ester (CFSE)-labeled cells. All analyses were performed using flow cytometry. The influence of MPA on apoptosis was dependent on the mechanism of cell activation and MPA concentration. MPA caused a decrease in the expression of lymphocyte surface antigens, CD3, CD8 and CD25. Its impact on the expression of CD4 and CD21 was negligible. Its negative influence on the expression of FoxP3 was dependent on cell stimulation. MPA inhibited lymphocyte proliferation. In conclusion, MPA inhibited the activity of stimulated canine lymphocytes by blocking lymphocyte activation and proliferation. The influence of MPA on the development of immune tolerance–expansion of Treg cells and lymphocyte apoptosis–was ambiguous and was dependent on the mechanism of cellular activation. The concentration that MPA reaches in the blood may lead to inhibition of the functions of the canine immune system. The applied panel of markers can be used for evaluation of the effects of immunosuppressive compounds in the dog.

## Introduction

Mycophenolic acid (MPA) is an active metabolite of mycophenolate mofetil (MMF), an immunosuppressive drug which was recently introduced to veterinary medicine. MPA blocks production of guanine nucleotides by non-competitive and reversible inhibition of inosine 5'-monophosphate dehydrogenase activity (IMPDH) [1, 2]. Its primary mechanism of action, inhibition of proliferation, reveals itself almost exclusively in activated lymphocytes [3, 4]. Literature data suggest that its effect goes beyond lymphocyte division arrest. Among others, it has been shown to selectively inhibit cell metabolic activity, decrease surface antigen expression and cytokine production and block differentiation into effector cells [3–10]. Previous studies have proven the efficacy of MPA in organ transplantation and autoimmune disease in humans. The use of MMF for the treatment of autoimmune diseases resistant to routine therapies is becoming more widespread also in the dog [11]. Its major advantages include lack of serious adverse side effects and low risk of toxicity [11]. In the veterinary literature there are suggestions of its application in myasthenia [12–14], immune-mediated hemolytic anemia (IMHA) [15–17], anaplastic anemia [18], immune-mediated thrombocytopenia [19, 20], pemphigus vulgaris, pemphigus foliaceus [21–23], inflammatory bowel disease (IBD) [24], glomerular diseases including glomerulonephritis [25, 26], immune-mediated arthritis [27] and necrotizing meningoencephalitis [28].

In humans therapeutic concentrations of MPA after oral administration vary between 1 μM (10^−3^ mol/m^3^) (0.32 mg/L) and 10 μM (3.2 mg/L) [29–32]. These values are similar to the ones obtained in dogs treated with MMF at a dose between 10 and 20 mg/kg [32, 33]. Administration of MMF to dogs at a dose of 7.5 mg/kg twice daily led in some animals to an increase in the maximal concentration of MPA in the blood to values exceeding 62.5 μM (20 mg/l) [32, 33]. Definitive recommendations for MMF dosing protocols are unavailable.

The action of immunosuppressive drugs can be evaluated by measurement of their target enzymes activity, but monitoring markers of immune system activity, such as, lymphocyte proliferation and apoptosis, or expression of lymphocyte surface antigens, indicates their true impact on the immune system [34]. Despite the influence of MPA and MMF on the activity of the immune system of humans and laboratory animals having been previously evaluated in multiple studies, data on the impact of MPA on immunity are still ambiguous [4, 35–37]. In the dog pharmacodynamics of MMF were assessed only by the measurement of IMPDH activity [38]. There are no published data on the influence of MPA on the canine immune system. Therefore there is a need for preliminary, preclinical studies evaluating its pharmacodynamic properties and assessing currently used MMF dosing protocols in the dog.

The aim of this study was to evaluate the influence of MPA on canine lymphocytes *in vitro*. The effects of MPA, at concentrations which are reached in the blood of treated animals, on lymphocyte proliferation, apoptosis and the expression of lymphocyte antigens: CD3, CD4, CD8, CD21, CD25, FoxP3 and PCNA were assessed.

## Materials and Methods

### Animals and blood samples

Twenty nine healthy, adult dogs, patients of 4 veterinary clinics in Warsaw, Poland, were included in the study after initial assessment which included anamnesis, physical examination and hematology. The inclusion criteria were: lack of clinical and laboratory signs of disease, no history of treatment or vaccination 2 weeks before blood sampling. The median age of the dogs was 6 years (range 2–10). All dogs were female neutered, 21 were mixed breed and eight were Labradors.

Peripheral blood samples were taken by cephalic venipuncture after 12 h fasting. Blood was anticoagulated with dipotassium ethylenediaminetetraacetic acid (K_2_-EDTA) or sodium heparin. K_2_-EDTA blood was used for hematology testing and heparinized samples were utilized for flow cytometric determination of the influence of MPA on peripheral blood mononuclear cells (PBMC) in culture. All analyses were performed at the Department of Pathology and Veterinary Diagnostics at the Faculty of Veterinary Medicine, Warsaw University of Life Sciences (WULS-SGGW), Warsaw, Poland.

Only excess peripheral blood (3 ml) collected for routine diagnostic tests was used for this study. The study was not subject to the Polish regulations on animal experiments valid at the time it was conducted as it did not cause any pain, suffering, distress or lasting harm to the animal and included only routine procedures performed under veterinary license. The study did not require a local ethics committee approval. The local ethics committee was informed about the design of the study and waived approval requirements (Local Ethical Committee for Animal Experiments at WULS-SGGW). The dogs' owners provided written, informed consent authorizing the use of the excess blood in this study. The dogs were required to fast for routine blood sampling; fasting was not required specifically for the purpose of this study.

### Hematology

Hematological analysis was performed in all dogs as a part of their initial evaluation. Complete blood count was done using an Abacus hematology analyzer (Diatron, Budapest, Hungary). Blood smears were examined with a CX21 light microscope (Olympus, Tokyo, Japan) after May-Grünwald Giemsa staining.

### Mycophenolic acid

MPA (M3536, Sigma-Aldrich, Saint Louis, USA) was dissolved in dimethyl sulfoxide (DMSO) (D2438, Sigma-Aldrich) in order to obtain a 0.1 M stock solution. The solution was then further diluted in DMSO and phosphate-buffered saline (PBS) (P4417, Sigma-Aldrich) and added to the complete culture medium. Cells were cultured in concentrations which MPA reaches in the plasma of treated patients [32, 33]. Final MPA concentrations were 1 μM, 10 μM or 100 μM. Control samples were cultured in the presence of DMSO–solvent control. The DMSO concentration remained constant in all the samples at 0.1% (volume/volume). All solutions were prepared aseptically on the day of the experiment.

### Culture of canine PBMC

Briefly, PBMCs were obtained from heparinized blood by density gradient centrifugation using Histopaque 1077 (10771, Sigma-Aldrich, specific gravity 1.077 g/ml) according to the manufacturer’s instruction. After, the PBMCs were harvested from the plasma Histopaque interface, the cells were washed with RPMI 1640 culture medium with L-glutamine (SH30027, HyClone, Thermo Fisher Scientific, Waltham, USA), and an antibiotic and antimycotic (penicillin 500 I.U./ml, streptomycin 50 μg/ml and neomycin 100 μg/ml) (P4083, Sigma-Aldrich) and the remaining erythrocytes were lysed by incubation with a 0.153 M tris(hydroxymethyl)aminomethane (TRIS) (T1503, Sigma-Aldrich) buffered 0.016 M ammonium chloride (A9434, Sigma-Aldrich) solution (pH 7.2) for 10 min on ice. Next, cells were washed with PBS and stained with a trypan blue dye (T8154, Sigma-Aldrich) to visualize dead cells according to a protocol described previously [39]. Sample cellularity and cell viability were assessed in a Thoma cell counting chamber. Samples designated for the determination of the proliferation indices were supravitally stained with carboxyfluorescein diacetate succinimidyl ester (CFSE) before culture. Next, all samples were washed three times with complete culture medium consisting of RPMI 1640, L-glutamine, 10% fetal bovine serum (FBS) (SV30160, HyClone, Thermo Fisher Scientific) and an antibiotic and antimycotic. PBMCs were placed in a 96-well flat-bottom plate (353072, Falcon, BD) at a concentration of 2x10^5^ cells in 200 μl of the culture medium per well and cultured for 72 h (37°C, 5% CO_2_) with or without MPA (various concentrations) and mitogens– 1 μg/ml concanavalin A (ConA) (C5275, Sigma-Aldrich) or 10 μg/ml phytohemagglutinin (PHA) (L1668, Sigma-Aldrich). Optimal concentrations of both mitogens, causing the strongest proliferation of cultured canine lymphocytes in conditions identical with the ones used in this study, were determined in a preliminary study (data not shown). Cell viability was reassessed after culture using trypan blue staining. The cultured cells were then used to evaluate the influence of MPA on the proliferation and apoptosis of lymphocytes, as well as antigen expression.

### CFSE labeling

Cell proliferation was assessed through dividing cell tracking (DCT) technique utilizing CFSE staining as described previously [40, 41]. CFSE is a dye which permanently binds to intracellular proteins. The reduction of its fluorescence in filial generations allows tracking of cell divisions [40]. Briefly, the 0.5 mM working solution of CFSE (21888, Sigma-Aldrich) was obtained by diluting 5 mM stock DMSO solution in 0.1% bovine serum albumin (BSA) (A7906, Sigma-Aldrich) in PBS. After evaluation of sample cellularity and cell viability 1x10^7^ cells were suspended in 1 ml of 0.1% BSA in PBS and stained in duplicates with CFSE at a final concentration of 5 μM for 10 min at 37°C in the dark. The process was stopped by adding 5 ml of cold complete culture medium and 5 min incubation on ice. Next, the samples were used for establishing a cell culture as described above. After culture, the ConA and PHA-stimulated samples were washed in a cold PBS solution and used to assess cell apoptosis. The unstimulated samples were suspended in the 1X Annexin V Binding Buffer (51-66121E, BD) and analyzed on the flow cytometer.

### Annexin V:PE and 7-AAD staining

Annexin V:PE Apoptosis Detection Kit I (559763, BD) containing Annexin V conjugated with phycoerythrin (PE) and 7-aminoactinomycin D (7-AAD) were used to distinguish non-apoptotic cells from cells in early apoptosis and late apoptosis/necrosis [42–44]. The CFSE-stained cells cultured with mitogens were labeled in duplicates according to the manufacturer’s instructions. Briefly, after washing in cold PBS solution, the cells were suspended in 100 μl of the 1X Annexin V Binding Buffer, 2.5 μl of Annexin V:PE and 2.5 μl of 7-AAD were added and the samples were incubated in the dark for 15 min at room temp. The incubation was stopped by addition of 100 μl of the 1X Annexin V Binding Buffer. Samples remained on ice until analysis.

### Immunofluorescence staining of lymphocyte antigens

#### CD3 / CD4 / CD8 and CD21

The influence of the MPA on the expression of lymphocyte surface antigens–CD3, CD4, CD8 and CD21 –was evaluated in the mitogen-stimulated cells using the following anti-canine mAbs [45–47]: mouse anti-CD3:FITC (clone CA17.2A12), rat anti-CD4:PE (clone YKIX302.9) and rat anti-CD8α:Alexa Fluor 647 (clone YCATE55.9)–triple color reagent (TC014, AbD Serotec) or mouse anti-CD21:PE–single color staining (clone CA2.1D6, MCA1781PE, AbD Serotec) (Table 1). The cultured cells were washed in the cytometric buffer. Next, 30% FBS in PBS was added to the samples to block the non-specific binding sites and the samples were incubated with the antibodies at room temp for 30 min. in the dark. Samples were then washed, resuspended in the cytometric buffer and analyzed on the flow cytometer. Isotype controls were used–mouse IgG1:FITC, rat IgG2a:PE and rat IgG1:Alexa Fluor 647 –antibody mix (TC022, AbD Serotec) or mouse IgG1:PE (MCA928PE, AbD Serotec).

**Table 1:** Characteristics of monoclonal antibodies used in immunofluorescense staining of lymphocyte antigens.

Antibody	Clone	Isotype	Host species	Antigen used to raise the antibody	Fluorochrome	Catalogue number	Supplier	Dilution
Anti-CD3	CA17.2A12	IgG1	Mouse	Canine affinity enriched TCR/CD3 membrane proteins isolated from thymocytes and the T cell line CLGL-90	FITC	TC014	AbD Serotec, UK	1:13
Anti-CD4	YKIX302.9	IgG2a	Rat	Canine ConA activated T-cell blasts	PE/FITC	TC014/MCA1038F	AbD Serotec, UK	1:13
Anti-CD8α	YCATE55.9	IgG1	Rat	Canine CD8 alpha chimeric human IgG1 Fc fusion protein	Alexa Fluor 647	TC014	AbD Serotec, UK	1:13
Anti-CD21	CA2.1D6	IgG1	Mouse	Canine CD21 –likely a homologue of human CD21	PE	MCA1781PE	AbD Serotec, UK	1:13
Anti-CD25	P4A10	IgG1	Mouse	Canine CD25, interleukin-2 receptor alpha chain	eFluor 660	50–0250	eBioscience, USA	1:20
Anti-FoxP3	FJK-16s	IgG2a	Rat	Murine FoxP3 protein	PE	12–5773	eBioscience, USA	1:20
Anti-PCNA	PC10	IgG2a	Mouse	Rat PCNA made in the protein A expression vector pR1T2T	FITC	MCA1558F	AbD Serotec, UK	1:10

Abbreviations: ConA, concanavilin A; FITC, fluorescein isothiocyanate; PE, phycoerythrin

#### CD4 / CD25 / foxP3

Tricolor flow cytometry was used to identify the influence of MPA on the population of ConA and PHA-stimulated regulatory T (Treg) cells and the expression of CD25 –a late activation marker. Surface antigens were stained using anti-canine mAbs (Table 1): rat anti-CD4:FITC (clone YKIX302.9, MCA1038F, AbD Serotec), mouse anti-CD25:eFluor 660 (clone P4A10, 50–0250, eBioscience, San Diego, USA) according to protocol of staining extracellular antigens described above [48, 49]. Next, forkhead box P3 (FoxP3) expression was evaluated using a cross-reactive anti-FoxP3:PE antibody as described previously by Biller et al. using a FoxP3/Transcription Factor Staining Buffer Set (00–5523, eBioscience) [48, 50]. Briefly, the samples were incubated overnight in the 1X Fixation/Permeabilization solution at 4°C in the dark. The samples were then washed in a 1X Permeabilization Buffer and incubated with the rat anti-FoxP3:PE mAb (clone FJK-16s, 12–5773, eBioscience) cross-reacting with dog cells at room temp. for 30 min (Table 1). After staining, the samples were washed again, suspended in the cytometric buffer and analyzed. Samples incubated with corresponding isotype were used as a negative control: rat IgG2a:FITC (MCA1212F, AbD Serotec), mouse IgG1:eFluor 660 (50–4714, eBioscience) and rat IgG2a:PE (12–4321, eBioscience).

#### PCNA

Lymphocyte proliferation was assessed by evaluation of PCNA expression after permeabilization of the cells with Leucoperm kit (BUF09, AbD Serotec) with methanol modification according to the manufacturer’s recommendations. PCNA is an auxiliary protein of the DNA polymerase δ the concentration of which increases at the end of the G1 phase of the cell cycle reaching its peak in S phase [51]. Briefly, the cells cultured with MPA and mitogens were washed with a cytometric buffer– 3% FBS and 0.1% NaN_3_ (S2002, Sigma-Aldrich) in PBS. After fixation of the cells by incubation with cold Leucoperm A reagent for 10 min. at 4°C, ice-cold absolute methanol was added and the cells were incubated for another 10 min. Next, the samples were washed with PBS and incubated with a cross-reactive murine anti-PCNA monoclonal antibody (mAb) (clone PC10, MCA1558F, AbD Serotec, Kidlington, United Kingdom) [52–55] conjugated with fluorescein isothiocyanate (FITC) (Table 1) and Leucoperm B reagent at room temp. for 30 min. in the dark. The samples were then washed, suspended in the cytometric buffer and analyzed. Cells incubated with a corresponding isotype control–mouse IgG2a:FITC (MCA929F, AbD Serotec) were used as a negative control.

### Flow cytometric analysis

Cytometrical analysis was performed using a FACSCanto II flow cytometer with an automated BD High Throughput Sampler (HTS) and FACSDiva 7.0 software (BD). The instrument was calibrated using BD FACSDiva CS&T Research Beads (655050, BD) and the Cytometer Setup and Tracking (CS&T) module in the BD FACSDiva software (BD). For multi-color staining compensation was set using cells single-positive for each color. The analysis was done within 1 h from cell staining. Lymphocytes were gated based on their light scatter properties. 20,000 cells from the lymphocyte gate were analyzed. For the CFSE assay the proliferation indices were calculated as a ratio of the percentage of cells proliferating after mitogen stimulation to the percentage of cells proliferating without stimulation. Cell apoptosis was evaluated by determination of the percentage of Annexin V:PE and 7-AAD positive cells. The expression of lymphocyte antigens was assessed through both the percentage of positive cells and the median fluorescent intensity (MFI) of the positive cells. Gates were set using isotype control antibodies.

### Statistical analysis

Results were presented as mean ± standard error of the mean (SEM). Data obtained from lymphocytes cultured with MPA and mitogens (ConA or PHA) were compared with controls (DMSO solvent control) cultured with the corresponding mitogen only. Statistical analysis was performed using Statistica 10.0 software (StatSoft, Tulsa, USA). The distribution of the data was assessed using the Shapiro-Wilk test. Skewed data were normalized using logarithmic transformation. The significance of differences was evaluated using a repeated measure analysis of variance (ANOVA) and a Tukey’s honestly significant difference (HSD) post-hoc test. Differences were considered significant when p<0.05 and highly significantly different when p<0.01 or p<0.001.

## Results

### MPA influence on lymphocyte viability and apoptosis

Viability of lymphocytes was assessed using trypan blue exclusion. Before and after culture with MPA, the viability of lymphocytes was above 95% for all the samples. Because this method cannot distinguish necrotic vs. apoptotic cells Annexin V/7-AAD staining to assess the percentage of early apoptotic and late apoptotic/necrotic cells using flow cytometry was performed.

The strategy for gating canine lymphocytes is presented in S1 Fig. The percentage of ConA-stimulated lymphocytes in early apoptosis after culture with MPA at the highest concentration (100 μM) was lower in comparison with the control and MPA at concentrations of 1 μM and 10 μM (p<0.05). Whereas, in samples cultured with PHA the percentage was higher in the concentration of 10 μM in comparison with the control and the concentration of 1 μM (p<0.05). A lower percentage of lymphocytes in late apoptosis was observed in the cells cultured with ConA and MPA at concentrations of 1 μM or 10 μM in comparison with the control (p<0.01) (S1 Table, S2 Fig). Differences in the percentage of dead cells between trypan blue exclusion test and Annexin V/7-AAD staining could have arised from the higher sensitivity of the latter.

### Influence of MPA on the percentage of CD3^+^, CD4^+^, CD8^+^ T and CD21^+^ B lymphocyte subpopulations

**Fig 1:**
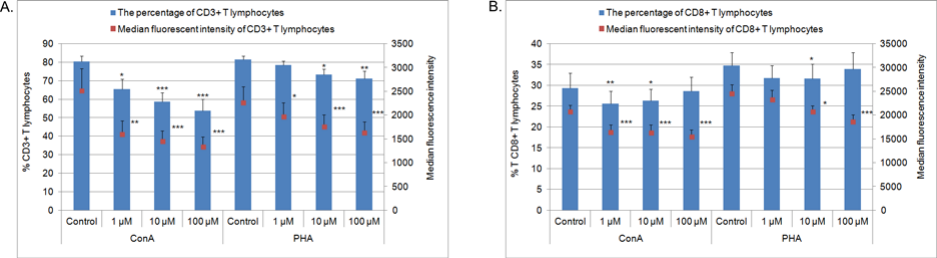
**The percentage and MFI of CD3^+^ (A) and CD8^+^ (B) lymphocytes** after 72 h culture of PBMC in a 37°C, 5% CO_2_ environment with mitogens–ConA or PHA and MPA at different concentrations (1 μM, 10 μM, 100 μM) or without MPA (solvent control– 0.1% DMSO) (n = 7). Mean ± SEM. *p<0.05, **p<0.01, ***p<0.001 in comparison with control. Abbreviations: ConA, concanavalin A; CFSE, carboxyfluorescein diacetate succinimidyl ester; DMSO, dimethyl sulfoxide; MFI, median fluorescence intensity; MPA, mycophenolic acid; PBMC, peripheral blood mononuclear cells; PCNA, proliferating cell nuclear antigen; PHA, phytohemagglutinin; SEM, standard error of the mean; μM, 10^−3^ mol/m^3^.

In cells cultured with MPA at all concentrations and ConA or PHA a lower percentage of CD3^+^ T lymphocytes (with the exception of the cells cultured with PHA and 1 μM MPA) and the MFI of the positive cells were observed in comparison with the control (p<0.05, p<0.01 or p<0.001, see Fig 1 and S2 Table for details), indicating that MPA action was concentration-dependent (S3 Fig).

The percentage of CD21^+^ B lymphocytes was higher in cells cultured with ConA and 1 μM MPA (p<0.05) and lower in samples cultured with PHA and MPA at concentrations of 10 μM (p<0.05) and 100 μM (p<0.01) in comparison with controls. Whereas, the impact of MPA on the MFI of ConA or PHA-stimulated CD21^+^ B lymphocytes was not observed in comparison with controls (S3 Table, S3 Fig).

There was no impact of MPA on the percentage of CD4^+^ T lymphocytes cultured with MPA and ConA or PHA in comparison with controls. Whereas, a lower MFI of CD4^+^ T lymphocytes was seen after culture with PHA and 100 μM MPA in comparison with the control (p<0.05) (S4 Table, S3 Fig).

For the influence of MPA on the percentage of CD8^+^ T lymphocytes, lower percentages of CD8^+^ T lymphocytes cultured with ConA and with MPA at concentrations of 1 μM (p<0.01) and 10 μM (p<0.05) and lymphocytes cultured with PHA and MPA at concentration of 10 μM (p<0.05) were noted in comparison with controls. The MFI of the CD8^+^ T lymphocytes cultured with ConA and MPA at all concentration was lower in comparison with the control (p<0.001). Whereas for the cells stimulated with PHA the MFI was lower in MPA at 10 μM (p<0.05) and 100 μM (p<0.001) in comparison with the control (Fig 1, S5 Table, S3 Fig).

The ratio of CD4^+^/CD8^+^ T lymphocytes cultured with ConA and 1 μM MPA or PHA and 10 μM MPA was higher in comparison with controls (p<0.05) (S6 Table, S3 Fig).

The percentage of CD4^+^CD8^+^ T lymphocytes cultured with ConA and MPA at the concentration of 100 μM was lower in comparison with the control (p<0.05) (S7 Table, S3 Fig).

### The influence of MPA on CD4^+^CD25^+^ T and CD4^+^CD25^+^FoxP3^+^ T lymphocyte subpopulations

**Fig 2:**
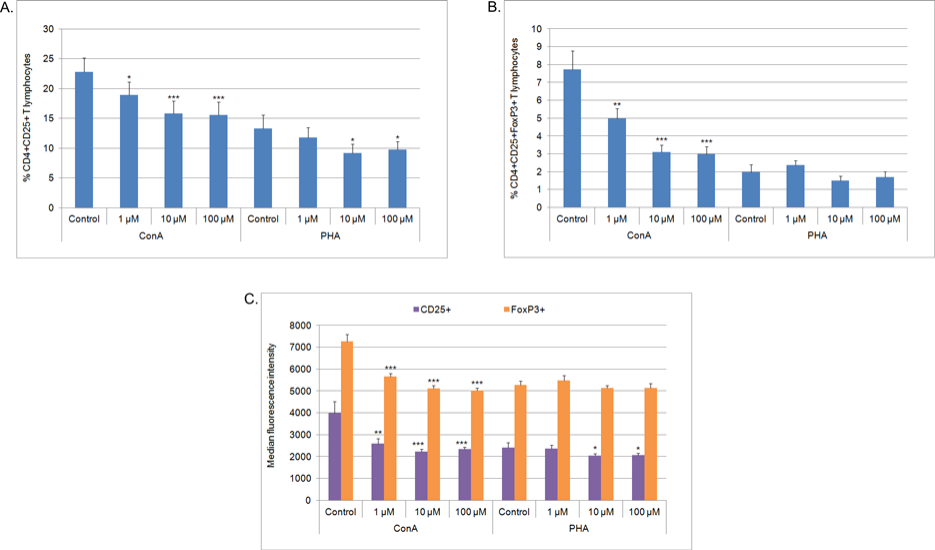
**The percentage of CD4**^**+**^**CD25**^**+**^ **(A), CD4**^**+**^**CD25**^**+**^**FoxP3**^**+**^ **(B) lymphocytes and MFI of FoxP3**^**+**^ **or CD25**^**+**^ **lymphocytes (C)** after 72 h culture of PBMC in a 37°C, 5% CO_2_ environment with mitogens–ConA or PHA and MPA at different concentrations (1 μM, 10 μM, 100 μM) or without MPA (solvent control– 0.1% DMSO) (n = 7). Mean ± SEM. *p<0.05, **p<0.01, ***p<0.001 in comparison with control.

Considering the influence of MPA on the percentage of CD4^+^CD25^+^ and CD4^+^CD25^+^FoxP3^+^ T lymphocytes, a marked, decrease was observed in cells cultured with ConA and MPA at all concentrations in comparison with the control (p<0.05, p<0.01 or p<0.001, see Fig 2 and S8 and S9 Tables for details). The action of MPA was strongest at 10 μM and 100 μM. The percentage of CD4^+^CD25^+^ T lymphocytes cultured with PHA and MPA at 10 μM and 100 μM was lower in comparison with the control (p<0.05). Whereas, the percentage of CD4^+^CD25^+^FoxP3^+^ T lymphocytes cultured with PHA was not affected by MPA in comparison with the control. MFI of CD25^+^ or FoxP3^+^ lymphocytes cultured with ConA and MPA at all concentrations was lower in comparison with the control (p<0.01 or p<0.001, see Fig 2 and S10 Table for details). In the case of PHA stimulation the MFI of CD25^+^ lymphocytes was lower in cells cultured with MPA at 10 μM and 100 μM in comparison with the control (p<0.05) (S3 Fig).

### Influence of MPA on lymphocytes proliferation

Proliferation of lymphocytes treated with MPA was assessed by PCNA expression and CFSE labeling.

**Fig 3:**
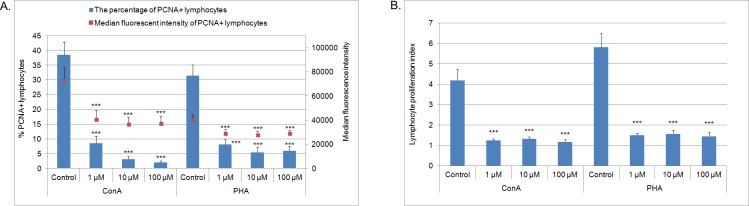
**The percentage and MFI of PCNA**^**+**^ **lymphocytes (n = 7) (A) and the proliferation index of CFSE-labeled lymphocytes (n = 8) (B)** after 72 h culture of PBMC in a 37°C, 5% CO_2_ environment with mitogens–ConA or PHA and MPA at different concentrations (1 μM, 10 μM, 100 μM) or without MPA (solvent control– 0.1% DMSO) Mean ± SEM. ***p<0.001 in comparison with control.

After culture with MPA at all concentrations, both, ConA or PHA-stimulated samples had a lower percentage of PCNA^+^ lymphocytes and MFI of PCNA^+^ lymphocytes in comparison with controls (p<0.001). The reduction in the percentage of PCNA^+^ lymphocytes was concentration-dependent in cells stimulated with ConA. In PHA-stimulated cells the percentage was lowest at 10 μM. Similar changes were observed for the MFI of the lymphocytes stimulated with ConA or PHA (Fig 3, S11 Table, S4 Fig).

The lymphocyte proliferation indices, assessed by CFSE labeling, were lower after a 3 day culture with MPA (at all concentrations) and mitogens (ConA or PHA) in comparison with controls (p<0.001) (Fig 3, S12 Table, S4 Fig). Taken together, these data indicated that MPA negatively regulates lymphocyte proliferation.

## Discussion

The results of this *in vitro* study showed that MPA, depending on the culture conditions, did not influence apoptosis or exhibit an anti- or proapoptotic effect. Moreover, MPA caused a decrease in the expression of lymphocyte surface antigens, such us, CD3, CD8 and CD25. Whereas, the influence of MPA on CD4 and CD21 expression was minimal. Depending on the activator used, MPA decreased or did not affect the expression of FoxP3 by lymphocytes. Finally, MPA inhibited proliferation of canine lymphocytes.

The results of this study indicated that the effect of MPA on lymphocyte apoptosis was dependent on the mechanism of cell activation and MPA concentration. In ConA-stimulated lymphocytes antiapoptotic action of MPA was observed. The influence of MPA on apoptosis of ConA-stimulated lymphocytes has not been previously evaluated. Discrepancies between our observation and the results of previous studies obtained using different activators might be explained by different mechanisms of cellular activation used in these studies or interspecies differences between the human, laboratory animals and the dog [3, 7, 9, 10, 56–62]. On the other hand, the results obtained on PHA-stimulated lymphocytes were in agreement with reports suggesting that MPA can show proapoptotic activity [3, 7, 56, 59–61]. As PHA has been shown to cause cell death on its own; induction of apoptosis might have been a consequence of a synergistic action of MPA and PHA [63]. Lack of a consistent increase in lymphocyte apoptosis was also previously reported and could have been explained by the design of our study [9, 10, 57, 58, 62, 64]. Quéméneur et al. observed that MPA induces cell apoptosis through S-phase arrest only in lymphocytes which were in the cell cycle prior to drug treatment [65]. It can be assumed that the low number of activated lymphocytes at the time of MPA treatment and mitogen stimulation could have prevented consequent manifestation of its proapoptotic activity in this study [65]. The data on the influence of MPA on lymphocyte apoptosis are still unclear and its proapoptotic action may be revealed only under certain conditions [9, 66].

A lower expression of CD3 and CD8 surface antigens after MPA treatment; measured as both, the percentage of positive cells and the MFI of antigens on positive cells was also observed in our study. This finding was in agreement with the reports showing that a longer incubation with MPA causes a decrease in the expression of surface antigens, including the expression of human CD3 [67, 68]. As MPA was previously shown to have an identical influence on the proliferation of activated CD4^+^ T, CD8^+^ T and CD21^+^ B lymphocytes [4, 6, 57] the drop in CD3 and CD8 expression was more likely a consequence of decreased production of these molecules. At the same time the synthesis of CD4 and CD21 might have been more resistant to MPA action.

This study has also indicated that MPA decreases the expression of an activation marker CD25 which was in agreement with the majority of previous studies performed on mitogen or antigen-stimulated human or rat lymphocytes [4, 36, 67, 69–72], as well as, many *in vivo* studies [36, 68, 71–74]. Our study has confirmed the findings of Thomson et al. and Prémaud et al. that the decrease in CD25 expression was partial and only partly depended on MPA concentration [68, 70]. It was also previously observed that the concentration that leads to complete inhibition of cell proliferation does not cause a full inhibition of the expression of activation markers, including CD25 [71, 75].

The way in which MPA inhibits the expression of surface antigens has not yet been fully elucidated. The decrease is suggested to be associated with decreased production of proteins due to a reduction in RNA synthesis and/or decreased protein G activity [76–78] rather than inhibition of protein glycosylation [29, 30, 79–81]. Alternatively, MPA instead of affecting the true number of molecules on the surface of the cells may have changed their structural conformation thereby reducing their ability to bind to monoclonal antibodies used in our study [77].

In our study, the percentage of double-positive CD4^+^CD8^+^ lymphocytes was lower only in the highest concentration of MPA in ConA-stimulated cells suggesting that it is not a sensitive marker of immunosuppression caused by MPA in the dog, which is contrary to previous observations made by Diaz-Romero et al. in rats after *in vitro* treatment of whole blood samples with calcineurin inhibitors [82]. This discrepancy could have been explained by a distinct mechanism of action of these drugs, as well as, species-specific differences (e.g. different roles and functions of the double-positive CD4^+^CD8^+^ cells in dogs and rats and/or higher resistance of this population of cells to immunosuppressive treatment in the dog).

The results of our study showed that MPA causes a concentration-dependant decrease in the expression of FoxP3 only in ConA-stimulated cells which was contrary to the majority of previous studies [9, 83–86] The decrease might have been a consequence of inhibition of ConA-induced Treg formation and proliferation [87, 88]. Whereas, in agreement with previous studies, a negative influence of MPA on the Treg population was not observed in PHA-activated cells [89–91]. The reason why MPA had a different impact on the Treg lymphocyte population depending on the mechanism of cellular activation remains unknown.

Significant inhibition of proliferation of mitogen-stimulated lymphocytes caused by MPA was detected by both assays used in this study, PCNA expression and CSFE labeling. This unsurprising finding was in agreement with multiple previous *in vitro* and *in vivo* studies performed in various settings in humans and laboratory animals [29, 30, 69, 71, 92–94]. As shown, lymphocyte proliferation was blocked by MPA at the lowest concentration used in this study in concordance with the previous findings of Allison and Eugui, who observed an inhibition of lymphocyte proliferation in nanomolar concentrations of MPA [95]. We observed a partially concentration-dependent decrease in proliferation only through the drop in PCNA expression which was in agreement with previous reports and suggested that the expression of PCNA may be a more sensitive marker of lymphocyte proliferation in the dog than the CFSE assay [29, 30, 71, 79, 96, 97].

The results of this study have shown that MPA at concentrations achieved in the blood of treated patients inhibits the activity of lymphocytes. Cell function significantly decreased at 1 μM and 10 μM. Although, the potency of the drug evaluated through a decrease in CD25 and PCNA expression was highest at 100 μM an increase in the MMF dose is not recommended due to an increased risk of adverse effects on the gastrointestinal tract which could lead to discontinuation of the therapy. *In vivo* the clinical effect of MPA treatment would likely be a sum of all the events caused by various MPA concentrations used in our study.

To the authors’ knowledge this is the first report evaluating the influence of MPA on canine lymphocytes. The mechanism of MMF action in the dog was previously evaluated only through IMPDH activity which does not reflect the drug’s impact on the activity of lymphocytes [34, 38]. Many MPA properties (e.g. inhibition of lymphocyte proliferation) were well studied in human and laboratory animals [4, 35–37]. At the same time many aspects of MPA’s action, despite numerous studies in these species, still remain unclear. Our study contributes to the clarification of these vague aspects of MPA action. Not infrequently responses to drugs can be very different between species, e.g. dogs and cats. Increased knowledge of the influence of MPA on canine immunity can be applied in veterinary pharmacotherapy and clinical immunology and is needed before the use of MMF in the dog becomes more widespread. Information regarding effects of various concentrations of MPA on canine lymphocytes was necessary for precise assessment of the effects of MMF treatment and evaluation of currently used MMF dosing protocols. Our findings will likely prove useful in future optimization of MMF dosing, allowing concurrent development of immune tolerance and minimalization of the risk of developing adverse effects of the drug, thus enabling maintenance of homeostasis in the immune system of the treated dogs. This is particularly important considering that the necessity of monitoring MMF treatment in dogs was previously suggested due to high interindividual variability of its pharmacokinetic properties [32, 98]. The results of this study may also play a significant role in planning of future *in vitro* and *in vivo* studies (including the selection of markers and activators) on the influence of MPA and other immunosuppressive drugs on the immune system of the dog. Our study is also a first report which has proven that flow cytometric assessment of PCNA expression is possible in the dog and should be considered a useful marker of lymphocyte proliferation in this species.

Despite, the novel and interesting findings, our study has some limitations. The fact that the study was conducted *in vitro* on PBMC is one of its drawbacks. It is generally considered that pharmacodynamic studies using whole blood culture are superior over studies on PBMC as they mimic to a greater extent *in vivo* conditions [35, 99, 100]. As information on the possibility of application of CFSE labeling in whole blood assays is lacking the use of PMBC allowed standardization of culture conditions in all tests in this study and simultaneous evaluation of lymphocyte proliferation by 2 different methods. At the same time an *in vitro* model using samples from healthy animals is superior to studies on animals with a particular autoimmune disease or tissues obtained from them and reflects the effect of the drug on immune cells which is unbiased by multiple confounding factors present *in vivo*. Accordingly, any studies on pharmacokinetics/pharmacodynamics (e.g. for antimicrobial drugs), toxicology, bioequivalence and tolerance studies must be provided on healthy animals [101–104]. The degree of variability in the expression of canine lymphocyte antigens (e.g. in the control) was similar to the one observed previously and was not unexpected given that the study included client-owned dogs, not research colony animals which have a more limited genetic pool [105].

The assessment of immunosuppression *in vitro* is routinely performed on mitogen-stimulated cells which better reflects the activation of the immune system in patients with immune-mediated diseases. At the same time mitogen stimulation can be considered a confounding factor which can, for instance, alter the expression of canine lymphocyte antigens [106]. Inclusion of an unstimulated control, both, after and without MPA treatment, could have been helpful in elucidating the more equivocal results of this study. Lack of the above is another limitation of this study. Although the mechanism of action of both mitogens used in this study, ConA and PHA, is quite similar, they produce significantly different effects in canine lymphocytes. ConA causes a higher expression of FoxP3 and CD25 than PHA. Whereas, PHA increases the percentage of apoptotic cells and the expression of CD4 and CD8 in comparison with ConA (data not shown). The use of both mitogens in this study allowed a more comprehensive evaluation of the lymphocytes.

The impact of MPA on cytokines, molecules playing a crucial role in stimulation and modulation of the immune response, was not evaluated which is also a limitation of this study [107]. The type and amount of cytokine production allows assessment of the immune system activity and immune response direction. A shift in the pattern of cytokine production may change the net immune response even when a deviation in the absolute or relative cell number does not occur. As cytokines may have different, frequently totally opposite, effects on immunity depending on the cells from which they originate, methods which simultaneously measure cytokine synthesis and assess the lineage of the producing cells are preferred, e.g. intracellular cytokine staining and subsequent multiparametric flow cytometric analysis [34, 108–110]. Despite reports suggesting that MPA preferentially blocks the Th2 cytokine response in humans [111–113] the exact effect of MPA on Th1/Th2 cytokine balance (cell-mediated versus humoral immunity) still needs to be elucidated [114, 115]. Future studies should evaluate the influence of MPA on Th1 cytokines (e.g. interferon-γ [IFN-γ] and interleukin-2 [IL-2]), Th2 cytokines (e.g. IL-4), and anti-inflammatory cytokines (e.g. IL-10 and transforming growth factor-β [TGF-β]) in the dog.

Further studies should also focus on the influence of MPA on the expression of activation markers other than CD25, including early activation markers, e.g., CD69 and CD71. Also assessment of the impact of MPA on NK and Th17 lymphocyte populations and Th17/Treg ratio are important in terms of evaluation of MPA pharmacodynamics in the dog.

Accurate assessment of lymphocyte activity in the dog is limited in comparison with humans and laboratory animals due to a small number of anti-canine antibodies and antibodies cross-reacting with this species, which hampers detailed cytokine profiling and evaluation of surface antigen expression. At the time the study was planned and conducted reagents necessary for evaluation of most of the above mentioned markers in the dog were not available (IL-10, TGF-β, CD69, CD71, as well as NK and Th17 cell markers) or exhibited weak cross-reactivity in commonly available assays (IL-4) [116]. Further studies *in vitro* and *in vivo* evaluating the influence of MPA on canine lymphocytes are warranted.

In conclusion, the activity of stimulated peripheral canine lymphocytes is not only blocked by MPA through inhibition of the proliferation of these cells *in vitro*, but the impact of MPA on the development of immune tolerance induced by lymphocyte apoptosis depended on the mechanisms of cellular activation and the concentration of MPA. Furthermore, MPA caused a decrease in the expression of surface antigens which leads to inhibition of lymphocyte activation and function. Depending on lymphocyte activation MPA could block the expansion of tolerogenic Treg lymphocytes. Finally, MPA at 1 μM and 10 μM caused a significant decrease in the function of canine lymphocytes *in vitro*.

## Supporting Information

S1 Fig Representative graphs demonstrating gating strategy of lymphocytes on a FSC vs. SSC dot plot after a 72 h culture of PBMC in a 37°C, 5% CO_2_ environment and PHA (mitogen). (TIF)

S2 Fig Representative dot plot demonstrating gating strategy of lymphocytes positive to Annexin V:PE and 7-AAD after a 72 h culture of PBMC in a 37°C, 5% CO_2_ environment and PHA (mitogen). (TIF)

S3 Fig Representative dot plots demonstrating gating strategy of lymphocytes expressing (isotype controls and test samples) CD3 and CD4 **(A)**, CD4 and CD8 **(B)**, CD21 **(C)**, CD4 and CD25 **(D)**, CD25 and FoxP3 **(E)** after a 72 h culture of PBMC in a 37°C, 5% CO_2_ environment and PHA (mitogen).
(TIF)

S4 Fig Representative histograms demonstrating gating strategy of lymphocytes expressing PCNA (isotype control and test sample) **(A),** proliferating CFSE-labeled lymphocytes (unstimulated control and test sample) **(B)** after a 72 h culture of PBMC in a 37°C, 5% CO_2_ environment and with or without PHA (mitogen).
(TIF)

S1 Table The percentage of lymphocytes in early (Annexin V:PE positive) and late (Annexin V:PE and 7-AAD positive) apoptosis after 72 h culture of PBMC in a 37°C, 5% CO_2_ environment with mitogens–ConA or PHA and MPA at 1 μM, 10 μM, 100 μM or without MPA (solvent control– 0.1% DMSO). Mean ± SEM (n = 8) *p<0.05, **p<0.01 in comparison with control; ^a^p<0.05 in comparison with 1 μM MPA; ^b^p<0.05 in comparison with 10 μM MPA
(PDF)

S2 Table The percentage and MFI of CD3^+^ T lymphocytes after 72 h culture of PBMC in a 37°C, 5% CO_2_ environment with mitogens–ConA or PHA and MPA at 1 μM, 10 μM, 100 μM or without MPA (solvent control– 0.1% DMSO). Mean ± SEM (n = 7) *p<0.05, **p<0.01, ***p<0.001 in comparison with control; ^a^p<0.05 in comparison with 1 μM MPA
(PDF)

S3 Table The percentage and MFI of CD21^+^ T lymphocytes after 72 h culture of PBMC in a 37°C, 5% CO_2_ environment with mitogens–ConA or PHA and MPA at 1 μM, 10 μM, 100 μM or without MPA (solvent control– 0.1% DMSO). Mean ± SEM (n = 7) *p<0.05, **p<0.01 in comparison with control; ^a^p<0.05, ^A^p<0.01 in comparison with 1 μM MPA
(PDF)

S4 Table The percentage and MFI of CD4^+^ T lymphocytes after 72 h culture of PBMC in a 37°C, 5% CO_2_ environment with mitogens–ConA or PHA and MPA at 1 μM, 10 μM, 100 μM or without MPA (solvent control– 0.1% DMSO). Mean ± SEM (n = 7) *p<0.05 in comparison with control; ^a^p<0.05, ^A^p<0.01 in comparison with 1 μM MPA
(PDF)

S5 Table The percentage and MFI of CD8^+^ T lymphocytes after 72 h culture of PBMC in a 37°C, 5% CO_2_ environment with mitogens–ConA or PHA and MPA at 1 μM, 10 μM, 100 μM or without MPA (solvent control– 0.1% DMSO). Mean ± SEM (n = 7) *p<0.05, **p<0.01, ***p<0.001 in comparison with control; ^a^p<0.05, ^A^p<0.01 in comparison with 1 μM MPA
(PDF)

S6 Table The CD4^+^/CD8^+^ T lymphocyte ratio after 72 h culture of PBMC in a 37°C, 5% CO_2_ environment with mitogens–ConA or PHA and MPA at 1 μM, 10 μM, 100 μM or without MPA (solvent control– 0.1% DMSO). Mean ± SEM (n = 7) *p<0.05 in comparison with control; ^a^p<0.05 in comparison with 1 μM MPA
(PDF)

S7 Table The percentage of CD4^+^CD8^+^ T lymphocytes after 72 h culture of PBMC in a 37°C, 5% CO_2_ environment with mitogens–ConA or PHA and MPA at 1 μM, 10 μM, 100 μM or without MPA (solvent control– 0.1% DMSO). Mean ± SEM (n = 7) *p<0.05 in comparison with control
(PDF)

S8 Table The percentage of CD4^+^CD25^+^ T lymphocytes after 72 h culture of PBMC in a 37°C, 5% CO_2_ environment with mitogens–ConA or PHA and MPA at 1 μM, 10 μM, 100 μM or without MPA (solvent control– 0.1% DMSO). Mean ± SEM (n = 7) *p<0.05, ***p<0.001 in comparison with control
(PDF)

S9 Table The percentage of CD4^+^CD25^+^FoxP3^+^ T lymphocytes after 72 h culture of PBMC in a 37°C, 5% CO_2_ environment with mitogens–ConA or PHA and MPA at 1 μM, 10 μM, 100 μM or without MPA (solvent control– 0.1% DMSO). Mean ± SEM (n = 7) **p<0.01, ***p<0.001 in comparison with control; ^a^p<0.05 in comparison with 1 μM MPA
(PDF)

S10 Table The MFI of FoxP3^+^ or CD25^+^ lymphocytes after 72 h culture of PBMC in a 37°C, 5% CO_2_ environment with mitogens–ConA or PHA and MPA at 1 μM, 10 μM, 100 μM or without MPA (solvent control– 0.1% DMSO). Mean ± SEM (n = 7) *p<0.05, **p<0.01, ***p<0.001 in comparison with control; ^a^p<0.05 in comparison with 1 μM MPA
(PDF)

S11 Table The percentage and MFI of PCNA^+^ lymphocytes after 72 h culture of PBMC in a 37°C, 5% CO_2_ environment with mitogens–ConA or PHA and MPA at 1 μM, 10 μM, 100 μM or without MPA (solvent control– 0.1% DMSO). Mean ± SEM (n = 7) ***p<0.001 in comparison with control; ^A^p<0.01 in comparison with 1 μM MPA
(PDF)

S12 Table Proliferation index of CFSE-labeled lymphocytes after 72 h culture of PBMC in a 37°C, 5% CO_2_ environment with mitogens–ConA or PHA and MPA at 1 μM, 10 μM, 100 μM or without MPA (solvent control– 0.1% DMSO). Mean ± SEM (n = 8) ***p<0.001 in comparison with control
(PDF)

## Abbreviations

7-AAD: 7-aminoactinomycin D
CFSE: carboxyfluorescein diacetate succinimidyl ester
ConA: concanavalin A
DMSO: dimethyl sulfoxide
FSC: forward scatter
MFI: median fluorescence intensity
MPA: mycophenolic acid
PBMC: peripheral blood mononuclear cells
PCNA: proliferating cell nuclear antigen
PHA: phytohemagglutinin
SEM: standard error of the mean
SSC: side scatter
μM: 10^−3^ mol/m^3^
