# Supplementary figures and images for: In Vitro Influence of Mycophenolic Acid on Selected Parameters of Stimulated Peripheral Canine Lymphocytes
Source: PLoS One. 2016 May 3;11(5):e0154429. doi: 10.1371/journal.pone.0154429 (PMC4854421; doi:10.1371/journal.pone.0154429)

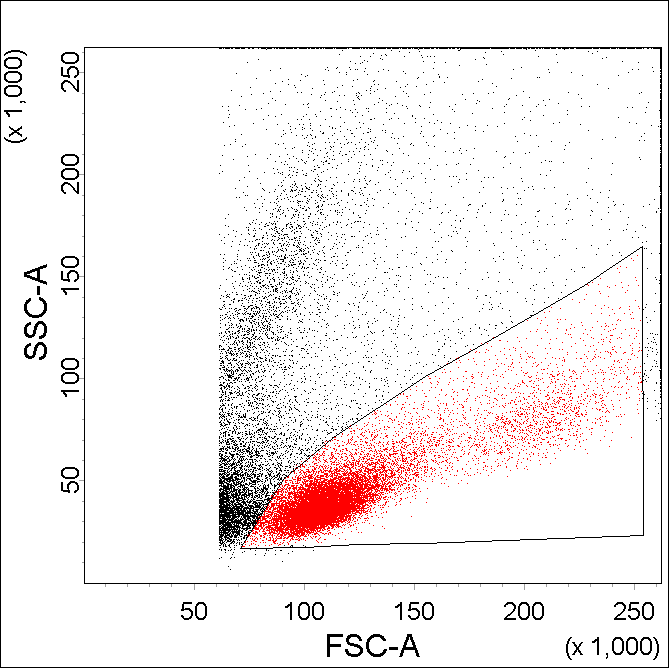

Supplement: S1 Fig — (TIF) [file pone.0154429.s001.tif]

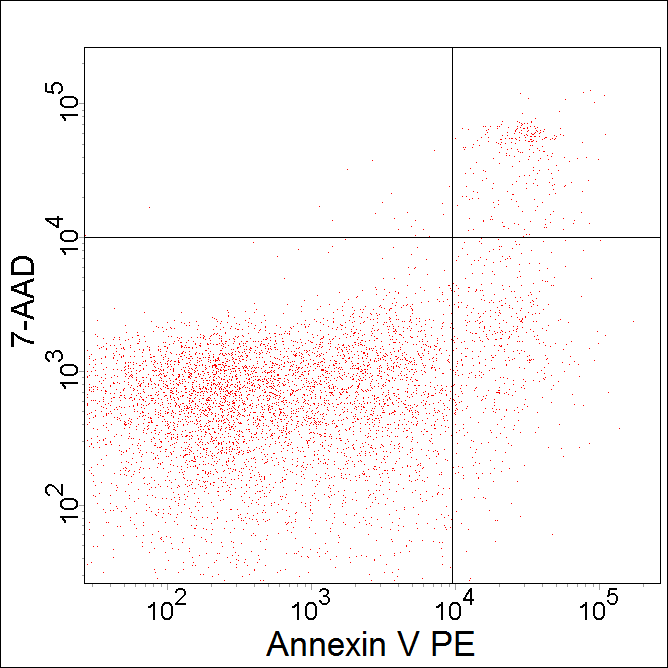

Supplement: S2 Fig — (TIF) [file pone.0154429.s002.tif]

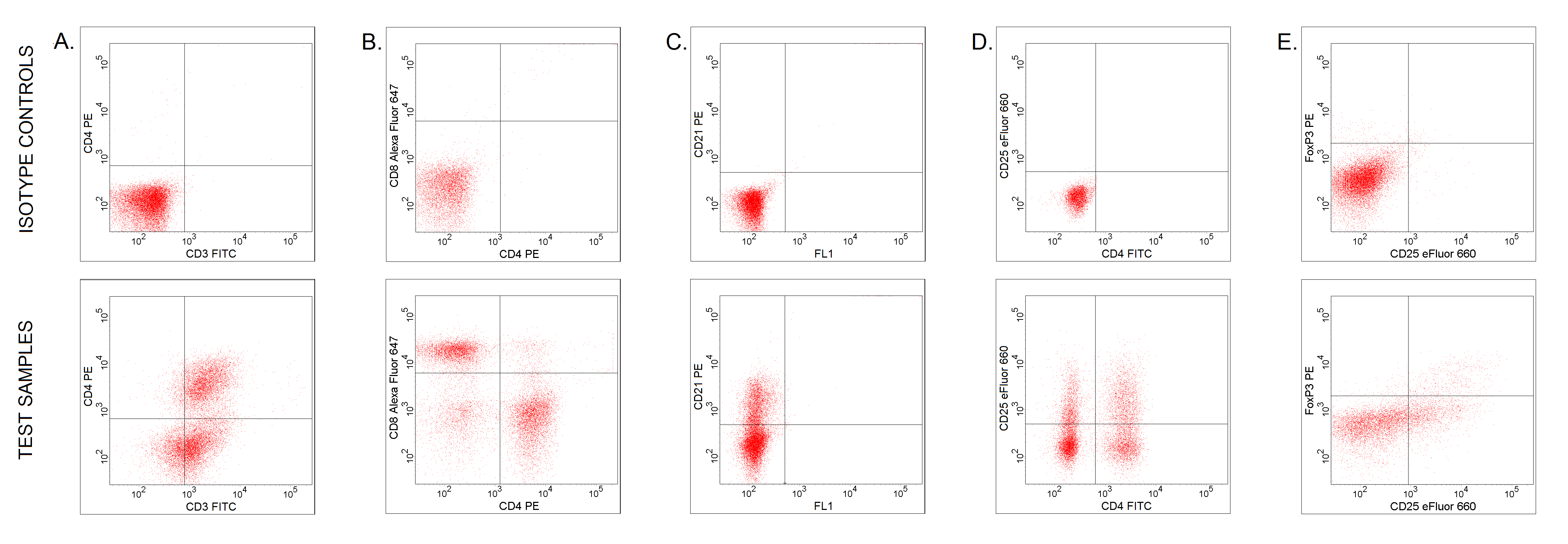

Supplement: S3 Fig — Representative dot plots demonstrating gating strategy of lymphocytes expressing (isotype controls and test samples) CD3 and CD4 (A), CD4 and CD8 (B), CD21 (C), CD4 and CD25 (D), CD25 and FoxP3 (E) after a 72 h culture of PBMC in a 37°C, 5% CO2 environment and PHA (mitogen). (TIF) [file pone.0154429.s003.tif]

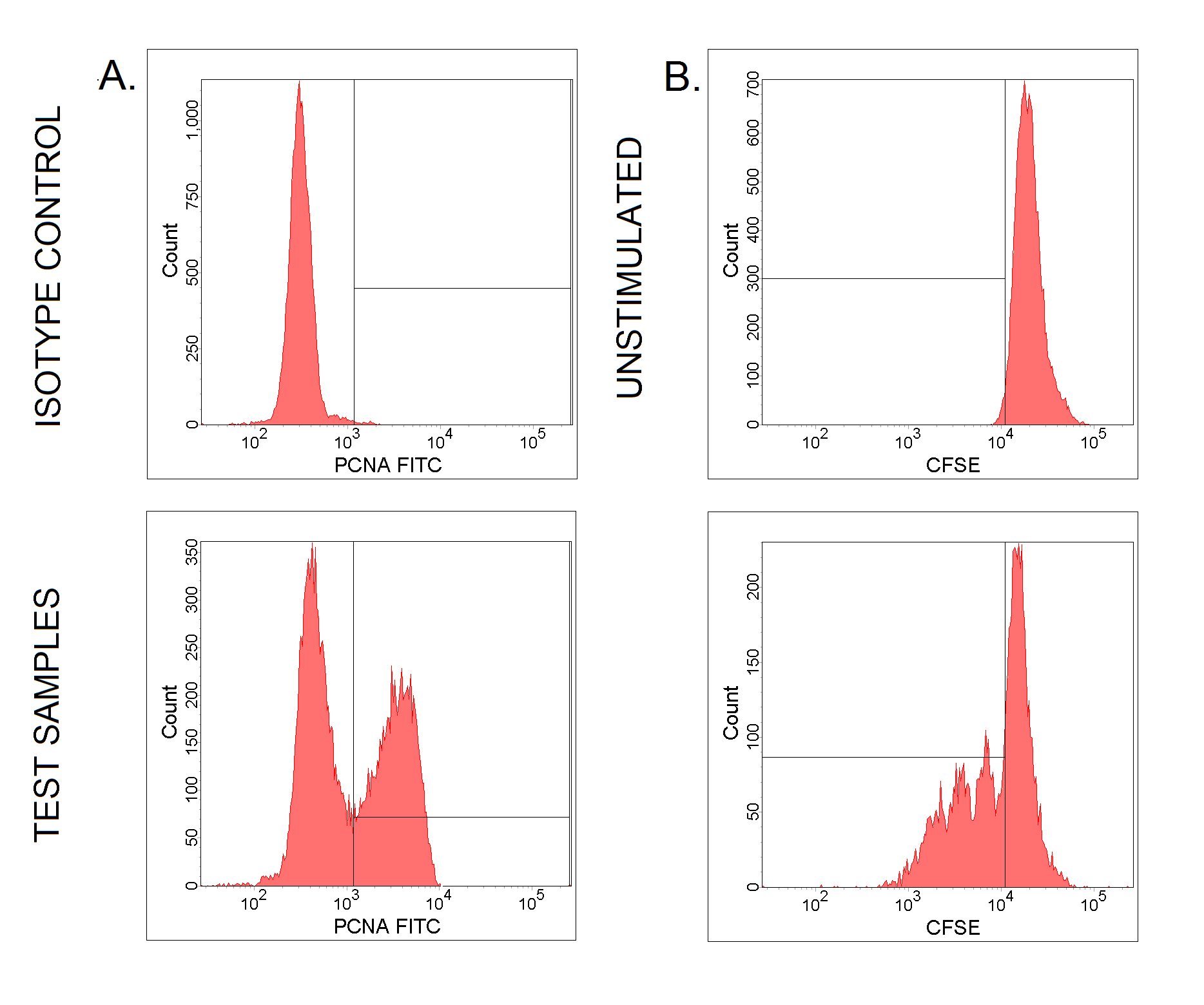

Supplement: S4 Fig — Representative histograms demonstrating gating strategy of lymphocytes expressing PCNA (isotype control and test sample) (A), proliferating CFSE-labeled lymphocytes (unstimulated control and test sample) (B) after a 72 h culture of PBMC in a 37°C, 5% CO2 environment and with or without PHA (mitogen). (TIF) [file pone.0154429.s004.tif]
