# Supplementary material for: In Vitro Influence of Mycophenolic Acid on Selected Parameters of Stimulated Peripheral Canine Lymphocytes
Source: PLoS One. 2016 May 3;11(5):e0154429. doi: 10.1371/journal.pone.0154429 (PMC4854421; doi:10.1371/journal.pone.0154429)
Supplement: S1 Table — Mean ± SEM (n = 8) *p<0.05, **p<0.01 in comparison with control; ap<0.05 in comparison with 1 μM MPA; bp<0.05 in comparison with 10 μM MPA (PDF) [file pone.0154429.s005.pdf]

S1 Table. The percentage of lymphocytes in early (Annexin V:PE positive) and late (Annexin V:PE and 7-AAD positive) apoptosis

after 72 h culture of PBMC in a 37°C, 5% CO_2 environment with mitogens – ConA or PHA and MPA at 1 µM, 10 µM, 100 µM or without MPA (solvent control – 0.1% DMSO). Mean ± SEM (n=8)

% apoptotic lymphocytes after culture with mitogens

MPA concentration	ConA		PHA	
	early phase	late phase	early phase	late phase
Control	5.63 ± 1.38	0.92 ± 0.11	4.88 ± 0.70	1.74 ± 0.30
1 µM	5.47 ± 1.27	0.49 ± 0.06**	5.11 ± 0.63	1.33 ± 0.34
10 µM	5.41 ± 1.27	0.53 ± 0.08**	6.26 ± 0.61*[,a]	1.86 ± 0.48
100 µM	3.96 ± 0.84*[,a,b]	0.66 ± 0.15	5.83 ± 0.95	2.21 ± 0.58

*p<0.05, **p<0.01 in comparison with control; [a]p<0.05 in comparison with 1 µM MPA; [b]p<0.05 in comparison with 10 µM MPA
