# Supplementary material for: In Vitro Influence of Mycophenolic Acid on Selected Parameters of Stimulated Peripheral Canine Lymphocytes
Source: PLoS One. 2016 May 3;11(5):e0154429. doi: 10.1371/journal.pone.0154429 (PMC4854421; doi:10.1371/journal.pone.0154429)
Supplement: S2 Table — Mean ± SEM (n = 7) *p<0.05, **p<0.01, ***p<0.001 in comparison with control; ap<0.05 in comparison with 1 μM MPA (PDF) [file pone.0154429.s006.pdf]

S2 Table. The percentage and MFI of $CD3^+$ T lymphocytes after 72 h culture of PBMC in a 37°C, 5% CO_2 environment with mitogens – ConA or PHA and MPA at 1 µM, 10 µM, 100 µM or without MPA (solvent control – 0.1% DMSO). Mean ± SEM (n=7)

**$CD3^+$ T lymphocytes after culture with mitogens**				
MPA concentration	ConA		PHA	
	% $CD3^+$	MFI	% $CD3^+$	MFI
Control	80.5 ± 2.8	2513 ± 469	81.6 ± 1.6	2267 ± 328
1 µM	65.4 ± 5.6*	1603 ± 277**	78.6 ± 2.2	1971 ± 293*
10 µM	58.7 ± 4.9***	1449 ± 218***	73.4 ± 2.9*	1758 ± 244***
100 µM	53.9 ± 6.0***, [a]	1331 ± 207***	71.0 ± 4.1**	1627 ± 237***, [a]

*p<0.05, **p<0.01, ***p<0.001 in comparison with control; [a]p<0.05 in comparison with 1 µM MPA
