# Supplementary material for: In Vitro Influence of Mycophenolic Acid on Selected Parameters of Stimulated Peripheral Canine Lymphocytes
Source: PLoS One. 2016 May 3;11(5):e0154429. doi: 10.1371/journal.pone.0154429 (PMC4854421; doi:10.1371/journal.pone.0154429)
Supplement: S3 Table — Mean ± SEM (n = 7) *p<0.05, **p<0.01 in comparison with control; ap<0.05, Ap<0.01 in comparison with 1 μM MPA (PDF) [file pone.0154429.s007.pdf]

S3 Table. The percentage and MFI of CD21⁺ T lymphocytes

after 72 h culture of PBMC in a 37°C, 5% CO2 environment with mitogens – ConA or PHA and MPA at 1 µM, 10 µM, 100 µM or without MPA (solvent control – 0.1% DMSO). Mean ± SEM (n=7)

$CD21^+$ B lymphocytes after culture with mitogens

MPA concentration	ConA		PHA	
	% $CD21^+$	MFI	% $CD21^+$	MFI
Control	15.8 ± 2.3	3701 ± 311	18.4 ± 2.5	3826 ± 351
1 µM	19.3 ± 2.4*	3892 ± 336	19.4 ± 2.9	3872 ± 346
10 µM	15.7 ± 2.2^{a}	3663 ± 351	15.3 ± 2.4^{*,A}	3787 ± 369
100 µM	13.3 ± 2.2^{A}	3522 ± 242	14.3 ± 2.7^{**,A}	3666 ± 286

*p<0.05, **p<0.01 in comparison with control; ^{a}p<0.05, ^{A}p<0.01 in comparison with 1 µM MPA
