# Supplementary material for: In Vitro Influence of Mycophenolic Acid on Selected Parameters of Stimulated Peripheral Canine Lymphocytes
Source: PLoS One. 2016 May 3;11(5):e0154429. doi: 10.1371/journal.pone.0154429 (PMC4854421; doi:10.1371/journal.pone.0154429)
Supplement: S4 Table — Mean ± SEM (n = 7) *p<0.05 in comparison with control; ap<0.05, Ap<0.01 in comparison with 1 μM MPA (PDF) [file pone.0154429.s008.pdf]

S4 Table. The percentage and MFI of CD4$^+$ T lymphocytes

after 72 h culture of PBMC in a 37°C, 5% CO_2 environment with mitogens – ConA or PHA and MPA at 1 μM, 10 μM, 100 μM or without MPA (solvent control – 0.1% DMSO). Mean ± SEM (n=7)

CD4$^+$ T lymphocytes after culture with mitogens				
MPA concentration	ConA		PHA	
	% CD4$^+$	MFI	% CD4$^+$	MFI
Control	45.2 ± 2.5	5917 ± 581	41.1 ± 2.5	7603 ± 651
1 μM	45.2 ± 2.0	6113 ± 602	43.2 ± 2.6	7979 ± 792
10 μM	45.2 ± 2.2	5563 ± 574	44.1 ± 2.7	7118 ± 480
100 μM	45.2 ± 2.9	5173 ± 485^a	41.2 ± 2.7	6426 ± 422^{*,A}

*$p<0.05$ in comparison with control; $^a p<0.05$, $^A p<0.01$ in comparison with 1 μM MPA
