# Supplementary material for: In Vitro Influence of Mycophenolic Acid on Selected Parameters of Stimulated Peripheral Canine Lymphocytes
Source: PLoS One. 2016 May 3;11(5):e0154429. doi: 10.1371/journal.pone.0154429 (PMC4854421; doi:10.1371/journal.pone.0154429)
Supplement: S5 Table — Mean ± SEM (n = 7) *p<0.05, **p<0.01, ***p<0.001 in comparison with control; ap<0.05, Ap<0.01 in comparison with 1 μM MPA (PDF) [file pone.0154429.s009.pdf]

S5 Table. The percentage and MFI of CD8$^+$ T lymphocytes after 72 h culture of PBMC in a 37°C, 5% CO_2 environment with mitogens – ConA or PHA and MPA at 1 μM, 10 μM, 100 μM or without MPA (solvent control – 0.1% DMSO). Mean ± SEM (n=7)

CD8$^+$ T lymphocytes after culture with mitogens				
MPA concentration	ConA		PHA	
	% CD8$^+$	MFI	% CD8$^+$	MFI
Control	29.3 ± 3.6	20743 ± 1377	34.8 ± 3.1	24620 ± 1727
1 μM	25.6 ± 2.9**	16429 ± 1477***	31.7 ± 3.1	23315 ± 1919
10 μM	26.3 ± 2.8*	16301 ± 1676***	31.6 ± 3.5*	20725 ± 1290*
100 μM	28.6 ± 3.4[a]	15480 ± 1444***	34.0 ± 3.9	18668 ± 1383***,[A]

*p<0.05, **p<0.01, ***p<0.001 in comparison with control; [a]p<0.05, [A]p<0.01 in comparison with 1 μM MPA
