# Supplementary material for: In Vitro Influence of Mycophenolic Acid on Selected Parameters of Stimulated Peripheral Canine Lymphocytes
Source: PLoS One. 2016 May 3;11(5):e0154429. doi: 10.1371/journal.pone.0154429 (PMC4854421; doi:10.1371/journal.pone.0154429)
Supplement: S6 Table — Mean ± SEM (n = 7) *p<0.05 in comparison with control; ap<0.05 in comparison with 1 μM MPA (PDF) [file pone.0154429.s010.pdf]

S6 Table. The $CD4^+/CD8^+$ T lymphocyte ratio

after 72 h culture of PBMC in a 37°C, 5% CO_2 environment with mitogens – ConA or PHA and MPA at 1 μM, 10 μM, 100 μM or without MPA (solvent control – 0.1% DMSO). Mean ± SEM (n=7)

**$CD4^+/CD8^+$ T lymphocytes ratio after culture with mitogens**		
MPA concentration	ConA	PHA
Control	1.72 ± 0.26	1.26 ± 0.17
1 μM	2.00 ± 0.29*	1.50 ± 0.24
10 μM	1.95 ± 0.29	1.56 ± 0.26*
100 μM	1.75 ± 0.31[a]	1.39 ± 0.27

*p<0.05 in comparison with control; [a]p<0.05 in comparison with 1 μM MPA
