# Supplementary material for: In Vitro Influence of Mycophenolic Acid on Selected Parameters of Stimulated Peripheral Canine Lymphocytes
Source: PLoS One. 2016 May 3;11(5):e0154429. doi: 10.1371/journal.pone.0154429 (PMC4854421; doi:10.1371/journal.pone.0154429)
Supplement: S7 Table — Mean ± SEM (n = 7) *p<0.05 in comparison with control (PDF) [file pone.0154429.s011.pdf]

S7 Table. The percentage of $CD4^+CD8^+$ T lymphocytes

after 72 h culture of PBMC in a 37°C, 5% CO2 environment with mitogens – ConA or PHA and MPA at 1 µM, 10 µM, 100 µM or without MPA (solvent control – 0.1% DMSO). Mean ± SEM (n=7)

*p<0.05 in comparison with control

% $CD4^+CD8^+$ T lymphocytes after culture with mitogens		
MPA concentration	ConA	PHA
Control	1.6 ± 0.2	1.5 ± 0.2
1 µM	1.3 ± 0.2	1.8 ± 0.5
10 µM	1.2 ± 0.2	1.7 ± 0.4
100 µM	1.1 ± 0.3*	1.7 ± 0.4

*p<0.05 in comparison with control
