# Supplementary material for: In Vitro Influence of Mycophenolic Acid on Selected Parameters of Stimulated Peripheral Canine Lymphocytes
Source: PLoS One. 2016 May 3;11(5):e0154429. doi: 10.1371/journal.pone.0154429 (PMC4854421; doi:10.1371/journal.pone.0154429)
Supplement: S8 Table — Mean ± SEM (n = 7) *p<0.05, ***p<0.001 in comparison with control (PDF) [file pone.0154429.s012.pdf]

S8 Table. The percentage of CD4$^+$CD25$^+$ T lymphocytes

after 72 h culture of PBMC in a 37°C, 5% CO_2 environment with mitogens – ConA or PHA and MPA at 1 µM, 10 µM, 100 µM or without MPA (solvent control – 0.1% DMSO). Mean ± SEM (n=7)

**% CD4$^+$CD25$^+$ T lymphocytes after culture with mitogens**		
MPA concentration	ConA	PHA
Control	22.8 ± 2.4	13.3 ± 2.3
1 µM	18.9 ± 2.2*	11.8 ± 1.7
10 µM	15.8 ± 2.1***	9.1 ± 1.5*
100 µM	15.6 ± 2.2***	9.7 ± 1.4*

*p<0.05, ***p<0.001 in comparison with control
