# Supplementary material for: In Vitro Influence of Mycophenolic Acid on Selected Parameters of Stimulated Peripheral Canine Lymphocytes
Source: PLoS One. 2016 May 3;11(5):e0154429. doi: 10.1371/journal.pone.0154429 (PMC4854421; doi:10.1371/journal.pone.0154429)
Supplement: S9 Table — Mean ± SEM (n = 7) **p<0.01, ***p<0.001 in comparison with control; ap<0.05 in comparison with 1 μM MPA (PDF) [file pone.0154429.s013.pdf]

S9 Table. The percentage of CD4$^+$CD25$^+$FoxP3$^+$ T lymphocytes after 72 h culture of PBMC in a 37°C, 5% CO_2 environment with mitogens – ConA or PHA and MPA at 1 μM, 10 μM, 100 μM or without MPA (solvent control – 0.1% DMSO). Mean ± SEM (n=7)

% CD4$^+$CD25$^+$FoxP3$^+$ T lymphocytes after culture with mitogens		
MPA concentration	ConA	PHA
Control	7.7 ± 1.0	2.0 ± 0.4
1 μM	5.0 ± 0.5**	2.4 ± 0.3
10 μM	3.1 ± 0.4***	1.5 ± 0.3[a]
100 μM	3.0 ± 0.4***,[a]	1.7 ± 0.3

**p<0.01, ***p<0.001 in comparison with control; [a]p<0.05 in comparison with 1 μM MPA
