# Supplementary material for: In Vitro Influence of Mycophenolic Acid on Selected Parameters of Stimulated Peripheral Canine Lymphocytes
Source: PLoS One. 2016 May 3;11(5):e0154429. doi: 10.1371/journal.pone.0154429 (PMC4854421; doi:10.1371/journal.pone.0154429)
Supplement: S10 Table — Mean ± SEM (n = 7) *p<0.05, **p<0.01, ***p<0.001 in comparison with control; ap<0.05 in comparison with 1 μM MPA (PDF) [file pone.0154429.s014.pdf]

**S10 Table. The MFI of FoxP3[+] or CD25[+] lymphocytes**
after 72 h culture of PBMC in a 37°C, 5% $CO_2$ environment with mitogens – ConA or PHA and MPA at 1 µM, 10 µM, 100 µM or without MPA (solvent control – 0.1% DMSO). Mean ± SEM (n=7)

**Mean fluorescence intensity of CD25[+] and FoxP3[+] after culture with mitogens**

| MPA concentration | ConA | | PHA | |
|---|---|---|---|---|
| | CD25+ | FoxP3+ | CD25+ | FoxP3+ |
| Control | 3986 ± 527 | 7272 ± 307 | 2400 ± 229 | 5263 ± 188 |
| 1 µM | 2603 ± 225** | 5652 ± 150*** | 2355 ± 164 | 5488 ± 227 |
| 10 µM | 2235 ± 106*** | 5108 ± 149*** | 2045 ± 100*,[a] | 5135 ± 115 |
| 100 µM | 2331 ± 104*** | 5021 ± 124*** | 2066 ± 93*,[a] | 5139 ± 199 |

*p<0.05, **p<0.01, ***p<0.001 in comparison with control; [a]p<0.05 in comparison with 1 µM MPA
