# Supplementary material for: In Vitro Influence of Mycophenolic Acid on Selected Parameters of Stimulated Peripheral Canine Lymphocytes
Source: PLoS One. 2016 May 3;11(5):e0154429. doi: 10.1371/journal.pone.0154429 (PMC4854421; doi:10.1371/journal.pone.0154429)
Supplement: S11 Table — Mean ± SEM (n = 7) ***p<0.001 in comparison with control; Ap<0.01 in comparison with 1 μM MPA (PDF) [file pone.0154429.s015.pdf]

S11 Table. The percentage and MFI of PCNA+ lymphocytes
after 72 h culture of PBMC in a 37°C, 5% CO_2 environment with mitogens – ConA or PHA and MPA at 1 µM, 10 µM, 100 µM or without MPA (solvent control – 0.1% DMSO). Mean ± SEM (n=7)

Lymphocyte proliferation after culture with mitogens				
MPA concentration	ConA		PHA	
	% PCNA+	MFI	% PCNA+	MFI
Control	38.5 ± 4.3	72856 ± 10787	31.4 ± 3.7	39786 ± 4714
1 µM	8.6 ± 2.4***	40445 ± 7794***	8.1 ± 1.6***	28988 ± 3253***
10 µM	3.2 ± 1.0***,A	36569 ± 5954***	5.3 ± 1.8***	27605 ± 3323***
100 µM	2.0 ± 0.6***,A	37281 ± 6185***	5.9 ± 1.5***	28755 ± 3041***

***p<0.001 in comparison with control; Ap<0.01 in comparison with 1 µM MPA
