# Supplementary material for: In Vitro Influence of Mycophenolic Acid on Selected Parameters of Stimulated Peripheral Canine Lymphocytes
Source: PLoS One. 2016 May 3;11(5):e0154429. doi: 10.1371/journal.pone.0154429 (PMC4854421; doi:10.1371/journal.pone.0154429)
Supplement: S12 Table — Mean ± SEM (n = 8) ***p<0.001 in comparison with control (PDF) [file pone.0154429.s016.pdf]

**S12 Table. Proliferation index of CFSE-labeled lymphocytes** after 72 h culture of PBMC in a 37°C, 5% $CO_2$ environment with mitogens – ConA or PHA and MPA at 1 µM, 10 µM, 100 µM or without MPA (solvent control – 0.1% DMSO). Mean ± SEM (n=8)

| | **Proliferation index after culture with mitogens** | |
|---|---|---|
| MPA concentration | ConA | PHA |
| Control | 4.18 ± 0.52 | 5.81 ± 0.66 |
| 1 µM | 1.24 ± 0.08*** | 1.50 ± 0.10*** |
| 10 µM | 1.31 ± 0.1*** | 1.55 ± 0.18*** |
| 100 µM | 1.15 ± 0.13*** | 1.44 ± 0.2*** |

***p<0.001 in comparison with control
